# 

**Published:** 1867-02

**Authors:** 


### CONTENTS.

### ORIGINAL CONTRIBUTIONS.

ART. VIII. A New Apparatus for Making Extension at the Ankle-Joint. By Julien S. Sherman, M.D., Chicago, ---------- 65

ART. IX. Cure of a Confirmed Opium-Eater.—Record of the Symptoms. By E. Andrews, M.D., Chicago, -------------------- 67

ART. X. Kernel of Corn in the Trachea.—Tracheotomy.—Expulsion of Foreign Body. By E. Andrews, M.D., Chicago, -------- 70

ART. XI. Pelvic Abscess. By Morton M. Eaton, M.D., Peoria, Ill., 71

ART. XII. Infantile Paralysis. By Chas. F. Taylor, M.D., N.Y., 74

ART. XIII. Remarks on Different Subjects. By N. Holton, M.D., Buda, Ill., ---------------------------------------- 76

### SELECTIONS.

Report on the Etiological and Pathological Relations of Epidemic Erysipelas, Spotted Fever, and Diphtheria. By N. S. Davis, M.D., Prof. of Principles and Practice of Medicine and of Clinical Medicine, in Chicago Medical College, ------------------ 80

New York Academy of Medicine, ------------------------------------ 90

The Medical Use of Electricity. By G. M. Beard, M.D., and A. D. Rockwell, M.D., New York, ---------------------------------------- 98

New York Medical Journal Association, ------------------------------ 103
Present Position of Aural Surgery.

### CLINICAL REPORTS.

Mercy Hospital Clinic:—Cardiac Disease with Œdema of the Lungs, etc. —Inflammation of Knee-Joint.—Clinic by Prof. N. S. Davis, ------ 107

### BOOK NOTICES.

Proceedings of the American Pharmaceutical Association, 14th Meeting, -- 111

Diphtheria; A Prize Essay. By E. S. Gaillard, M.D., Richmond, Va., -- 111

On the Relations which Electricity sustains to the Causes of Disease. By S. Littell, M.D., Philadelphia, ---------------------------------- 112

Reduction of Inverted Uteri. By Thomas Addis Emmet, M.D., ---------- 113

Clinical Observations on Functional Nervous Disorders. By C. H. Jones, 113

Infantile Paralysis, and the Attendant Deformities. By Chas. F. Taylor, 113

Notes on Epidemics. By Francis Edmund Anstie, M.D., F.R.C.P., ------ 113

The Common Nature of Epidemics, etc. By Southwood Smith, M.D., --- 114

Lessons upon the Diagnosis and Treatment of Surgical Diseases. By Prof. Velpeau, ------------------------------------------------------- 114

### EDITORIAL.

American Medical Association, --- 114

Illinois State Medical Society, ---- 116

Rush Med. College Commencement, 116

Chicago Med. Coll. Commencement, 117

Physician's Pocket Record, ------ 117

Books Not Received, ------------ 117

Notes upon General Science, ----- 118

Mortality for the Month of Jan'y, 120

Metallic Spectacles, ------------- 120

The Headquarters of Drunkenness, 121

Poisoned Bread at Winona, Ill., -- 121

A Remarkable Solvent, --------- 122

White Paste, ------------------ 122

New Instrument for Subcutaneous Injections, -------------------- 122

The Paris Exposition, ----------- 123

Cholera and Quarantine, -------- 123

The Gain in the Average Duration of Human Life, -------------- 123

International Med. Cong. of Paris, 123

Eustachian Tube normally closed except in Deglutition, --------- 124

Insufflation of Medicated Powders in Gleet, -------------------- 125

Prof. Unger, on Egyptian bricks, - 125

Rapidity of Nerve Action, ------- 126

